# Central Role of Maladapted Astrocytic Plasticity in Ischemic Brain Edema Formation

**DOI:** 10.3389/fncel.2016.00129

**Published:** 2016-05-13

**Authors:** Yu-Feng Wang, Vladimir Parpura

**Affiliations:** ^1^Department of Physiology, School of Basic Medical Sciences, Harbin Medical UniversityHarbin, China; ^2^Department of Neurobiology, University of Alabama at BirminghamBirmingham, AL, USA

**Keywords:** aquaporin-4, astrocytes, brain edema formation, glial fibrillary acidic protein, functional plasticity, hydromineral balance, ischemic stroke, morphological plasticity

## Abstract

Brain edema formation and the ensuing brain damages are the major cause of high mortality and long term disability following the occurrence of ischemic stroke. In this process, oxygen and glucose deprivation and the resulting reperfusion injury play primary roles. In response to the ischemic insult, the neurovascular unit experiences both intracellular and extracellular edemas, associated with maladapted astrocytic plasticity. The astrocytic plasticity includes both morphological and functional plasticity. The former involves a reactive gliosis and the subsequent glial retraction. It relates to the capacity of astrocytes to buffer changes in extracellular chemical levels, particularly K^+^ and glutamate, as well as the integrity of the blood-brain barrier (BBB). The latter involves the expression and activity of a series of ion and water transport proteins. These molecules are grouped together around glial fibrillary acidic protein (GFAP) and water channel protein aquaporin 4 (AQP4) to form functional networks, regulate hydromineral balance across cell membranes and maintain the integrity of the BBB. Intense ischemic challenges can disrupt these capacities of astrocytes and result in their maladaptation. The maladapted astrocytic plasticity in ischemic stroke cannot only disrupt the hydromineral homeostasis across astrocyte membrane and the BBB, but also leads to disorders of the whole neurovascular unit. This review focuses on how the maladapted astrocytic plasticity in ischemic stroke plays the central role in the brain edema formation.

Ischemic stroke is a major cause of death and disability, largely because of brain edema formation (Lackland et al., 2014). The brain edema formation mainly results from oxygen and glucose deprivation and reperfusion injury as well as a series of secondary events (Rutkowsky et al., 2011; O’Donnell et al., 2013). These events cause disturbance of hydromineral balance in the neurovascular unit (Kempski, 2001). At the early stage of ischemic insults, injured neurons, glial and endothelial cells experience cytotoxic cell swelling due to abnormal transport of ion and water across cell membranes in the gray matter. Prolonged ischemia and reperfusion injury result in vasogenic edema in the white matter because of increased permeability and destruction of the blood-brain barrier (BBB) and hydromineral retention in extracellular space (Castillo, 2000). The edema can lead to high intracranial pressure, cerebral herniation and death (Khanna et al., 2014) and thus becomes a focus of studies on ischemic stroke.

Accumulating evidence suggests that brain edema is a continuous process modulated by the plastic changes in astrocyte structures and functions that are associated with or can be directly attributed to GFAP, water channel protein AQP4 and their associated ion-transport proteins. This review focuses on the causal relationship between maladapted astrocytic plasticity and brain edema formation during ischemic stroke.

## Astrocytic Morphological Plasticity and Brain Edema Formation

Astrocytic endfeet cover more than 90% of brain capillaries to participate in blood-brain barrier (BBB) formation and wrap around neurons to modulate neuronal activity (Jukkola and Gu, 2015). This spatial structural feature allows astrocytes to mediate the communication and volume transmission between brain parenchyma and the blood (Vargová and Syková, 2014). Thus, when astrocyte processes expand or retract from neurons and blood vessels, neural activity and brain volume change dramatically (Nico and Ribatti, 2012), disorders of which constitute a basis of brain edema formation.

### Reactive Gliosis

In ischemic stroke, astrocytes undergo a dual morphological plasticity with strong temporal and spatial features. Reactive astrogliosis or elongation of astrocyte processes occurs under hypoxia in primary culture of astrocytes and in ischemic rat brains (Yang et al., 2011b). The reactive gliosis occurs at the initial stage of ischemic stroke or in the penumbra of infarction in severe infarction (Günther et al., 2005; Yang et al., 2011a), and is the main component of cytotoxic edema (Mori et al., 2002). On the one hand, gliosis can buffer damaging effects of ischemia by increasing astrocyte absorption of glutamate, K^+^ and inflammatory cytokines (Pekny et al., 2014) and separate the necrotic and healthy brain tissues clearly at the penumbra to prevent the expansion of infarct zone (Claus et al., 2013). On the other hand, uncontrolled gliosis can trigger maladaptation of astrocytic plasticity.

### Glial Retraction

**Figure 1:**
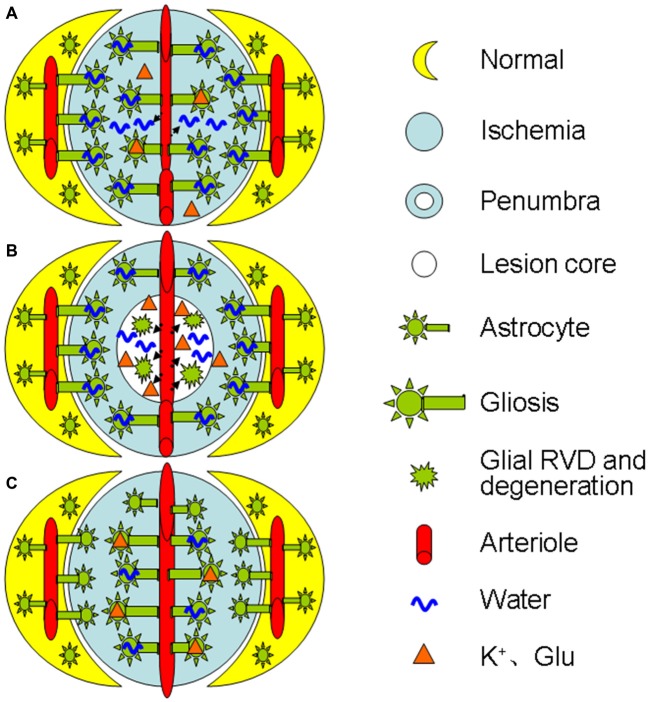
**Diagrammatic drawing of the spatiotemporal features of astrocytic plasticity in ischemic stroke (A–C).** Spatiotemporal features of astrocytic involvement in ischemic stroke in the early stage (0–24 h) of a mild stroke **(A)**, middle stage (>24–72 h, **B**) and the late stage **(C)**, respectively. The abbreviations are, Glu, glutamate; RVD, regulatory volume decrease.

As ischemia progresses, glial retraction occurs as indicated by the fragmentation of glial fibrillary acidic protein (GFAP) in the infarct zone in parallel with gliosis at the penumbra area or following the initially adaptive gliosis (Abrahám et al., 2003). This dual morphologic plasticity is in agreement with the finding that cerebral cytotoxic edema in prolonged ischemia becomes less severe than that at the initial stage of the middle cerebral artery occlusion in rats (Lu et al., 2011). This reaction can disrupt the structural integrity of the BBB directly and worsen the ischemic stress (Frydenlund et al., 2006; Steiner et al., 2012) by the following approaches: (1) In the lesion core, the swollen astrocytes can release K^+^, Cl^−^, and organic osmolytes including glutamate during a regulatory volume decrease (RVD; Quesada et al., 1998; Cardin et al., 1999; Ernest et al., 2005); (2) The RVD also reduces the absorption of excess K^+^ and glutamate produced during ischemia-evoked cortical spreading depolarization (Dreier, 2011; Seidel et al., 2016); and (3) Moreover, the RVD can cause cellular degeneration and metabolic silence (van der Zijden et al., 2008) as well as disruption of BBB integrity and leakage of blood components into the brain (Mdzinarishvili et al., 2012). As a result, extracellular osmolality increases, more water moves into the extracellular space from both blood and intracellular space, and brain edema forms. Clearly, ischemic challenge can elicit time- and space-dependent maladaptation of astrocytic morphological plasticity and contributes to the brain edema formation. Figure 1 is a diagrammatic drawing of the spatiotemporal features of astrocytic plasticity in ischemic stroke.

## Astrocytic Functional Plasticity and Edema Formation

Along with morphological plasticity, astrocytes also possess functional plasticity in brain hydromineral homeostasis by changing the expression of ion transport molecules. The molecules that are involved in this astrocytic functional plasticity in the edema formation include GFAP (Abrahám et al., 2003), AQP4 (Wang and Hatton, 2009), Na^+^, K^+^, 2Cl^−^ and water cotransporter (NKCC)1 (Hertz et al., 2014), sulfonylurea receptor 1-transient receptor potential melastatin4 channel (Karschin et al., 1998), sodium pump (Illarionova et al., 2010), glutamine synthetase (Wang et al., 2013b), glutamate-aspartate transporter (Sullivan et al., 2007; Gottipati et al., 2015), and glutamate transporter-1 (GLT-1) (Afadlal et al., 2014; Mogoanta et al., 2014) in addition to those that are involved in GABA (Wang et al., 2013a) and glutamate metabolism (Wang et al., 2013b). Among these molecules, GFAP is the leading molecule that influences the spatial localization of other molecules.

### GFAP and Edema

GFAP has long been known as the major cytoskeletal element of astrocytes, and assembling of its monomers or disassembling its filaments can largely determine if astrocyte processes expand or retract under environmental challenges (Barreto et al., 2012; Hol and Pekny, 2015). A scaffolding role of GFAP in astrocytic plasticity has been extensively explored in studying the effects of dehydration and lactation on supraoptic astrocyte activity as previously reviewed (Wang and Zhu, 2014). Consistently, single-walled carbon nanotubes cause an increase in astrocyte uptake of extracellular glutamate by increasing glutamate-aspartate transporter on cell surface, which results from an increase in GFAP filaments (Gottipati et al., 2015). Thus, the expansion of GFAP filaments in astrocyte processes pulls these associated molecules into the vicinity of neurons as shown in the distribution of AQP4 and glutamine synthetase in rats (Wang and Hatton, 2009). Consequently, astrocytes can more efficiently buffer neurochemical changes in the extracellular space around neurons.

Under ischemic challenges, the expression pattern of GFAP changes dramatically during reactive gliosis or retraction. Accumulation of GFAP in reactive astrocytes is a characteristic pathological feature of ischemic brain injury (Kalaivani et al., 2014). Following global cerebral ischemia, a tendency towards GFAP elevation was noticed after 24 h and a significant increase was observed 7 days later (Sulkowski et al., 2002). Moreover, the timing and magnitude of GFAP expression have dramatic regional variations relative to the infarct core (Abrahám et al., 2003). In contrast to the strong expression of GFAP within the penumbral tissues, GFAP staining in the lesion core area is significantly lower (Shen et al., 2010; Bazan et al., 2012), suggesting glial retraction. Correspondingly, the expression of other molecules also exhibit similar time- and region-specific features as exemplified in glutamate metabolism and ion transport activity.

### Glutamate Transport and Edema

Glutamate uptake by astrocytes prevents elevation of excitotoxic glutamate in the brain’s extracellular space and is a critical determinant of neuronal survival and cytotoxic edema in the ischemic penumbra (Hansson et al., 2000; Uckermann et al., 2004). In parallel with the change in GFAP expression, glutamate transport proteins also exhibit dual expression in a similar spatiotemporal order. For instance, the decreased GLT-1 experssion in the infarct zone during acute ischemic phase (Rebel et al., 2005; Ketheeswaranathan et al., 2011) can partially account for the increased glutamate levels (Yang et al., 2010) and the metabolic silence in the lesion core (van der Zijden et al., 2008), a result of glutamate excitotoxicity. In contrast, 3–7 days after the ligation of bilateral common carotid arteries in rats, GLT-1 expression is increased significantly (Yatomi et al., 2013). This change is coincident with astrocyte/GFAP extension into the lesion core (Shen et al., 2010), which meets the demand of removing excess extracellular glutamate, but at the expense of glial scar formation (Yang et al., 2010).

### Ion Transport and the Edema

Cytotoxic astrocyte swelling largely depends on osmotic gradients built by changes in ion transport activity, which involves NKCC1, inward rectifier K^+^ channel (Kir) 4.1 and sodium pump. Astrocyte NKCC1 is activated by exposure to high levels of extracellular K^+^ and to the regulatory volume increase in cells shrunken in response to hypertonic challenge (Larsen et al., 2014), an extracellular environment existing in the lesion core (van der Zijden et al., 2008; Yang et al., 2010). Increases in extracellular K^+^ levels, known to increase GFAP expression (Wang and Hatton, 2009), also increase Ca^2+^ influx and intracellular cAMP levels during stroke. The Ca^2+^ influx induces an increase in intracellular Ca^2+^ levels and the activation of NKCC1 and Kir4.1 (Song et al., 2015); increased cAMP level causes AQP4 phosphorylation and increases membrane water permeability (Song and Gunnarson, 2012). Thus, synergistic changes in the activity of diverse ion transport proteins with GFAP expression build the osmotic gradients.

## Pivotal Roles of AQP4 in Ischemia-Elicited Astrocyte Plasticity

Among many GFAP-associated molecules, AQP4 is distinguished not only by its water permeability but also by its role of a “cotransporter” of osmolytes. AQP4 is colocalized or assembled with the transient receptor potential vanilloid channel 4 (Jo et al., 2015), gap junction protein Cx43 (Nicchia et al., 2005), Kir4.1/Kir5.1 (Lichter-Konecki et al., 2008), GLT-1 (Mogoanta et al., 2014), metabotropic glutamate receptor 5, and sodium pump (Illarionova et al., 2010). AQP4 and these ion transport proteins are mainly colocalized to astrocytic endfeet and exhibit strong functional correlation. For instance, deletion of AQP4 interferes K^+^ (Strohschein et al., 2011) and glutamate uptake (Nagelhus and Ottersen, 2013) while significantly decreasing GFAP expression in perivascular processes of astrocytes (Zhou et al., 2008). This molecular association likely reflects the demands of maintaining intracellular osmotic homeostasis via synergistically changing osmotic pressure and water transport.

### Temporal Features

Following the occurrence of stroke, AQP4 expression presents two peaks despite the time variations among individual observations. The first peak occurs at the initial stage of stroke; the second peak appears days after reperfusion and during recovery. As shown in mice (Ribeiro Mde et al., 2006) and rats (Fang et al., 2016), there are two peaks of maximal hemispheric swelling that respectively occur at 1 h and at 48 h after the onset of ischemia and are accompanied with synergistic increases in AQP4 expression (Ribeiro Mde et al., 2006). Moreover, AQP4 at the infarct zone is found to be lower at 24 h and higher at 72 h than that at the non-occluded areas (Zeng et al., 2012). This biphasic AQP4 expression is consistent with the internalization and decomposition of AQP4 at 3 h after the onset of ischemia (Huang et al., 2013). Clearly, AQP4 expression is temporally synergistic with GFAP/astrocytic morphological plasticity.

### Spatial Features

In mice with 1 h transient focal brain ischemia, AQP4 expression is significantly increased at astrocyte endfeet in the lesion core initially and it increases in whole astrocytes at the border of lesion at 48 h (Ribeiro Mde et al., 2006). Moreover, the most affected part of the cortex loses perivascular AQP4 but shows a partial recovery toward 72 h of reperfusion; however, the cortical border zone differs from the lesion core by showing no loss of perivascular AQP4 at 24 h but rather a slight increase (Frydenlund et al., 2006). These region-specific changes indicate that AQP4 plays different role, depending on the regions, consistent with the general alteration in GFAP expression and astrocytic morphological plasticity.

**Figure 2:**
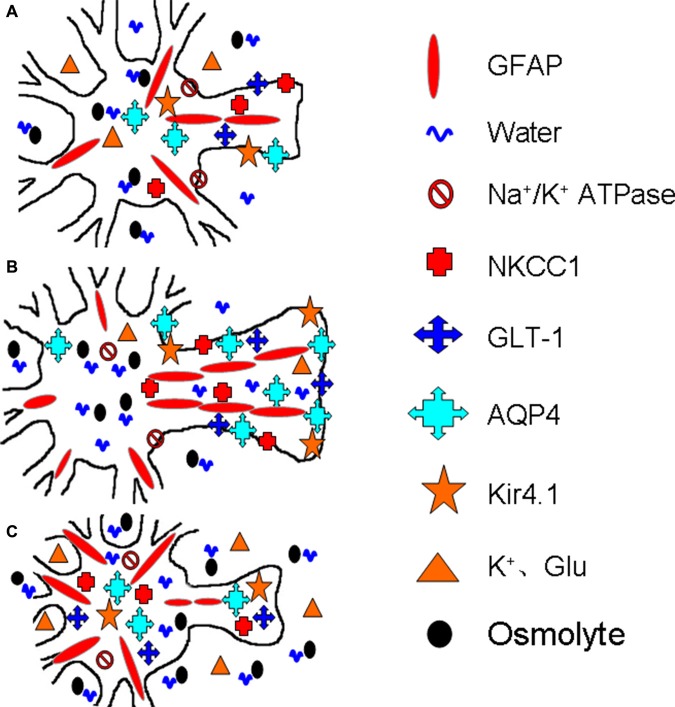
**Diagrammatic drawing of the astrocytic functional plasticity in ischemic stroke (A–C).** The functional plasticity at different loci relative to the infarct core: normally irrigated tissues **(A)**, the penumbra **(B)** and the lesion core **(C)**, respectively. The abbreviations are, AQP4, Aquaporin-4; GFAP, glial fibrillary acidic protein; GLT-1, glutamate transporter-1; Kir4.1, inward rectifier K^+^ channel 4.1; NKCC1, Na^+^, K^+^, 2Cl^−^ and water co-transporter. Others refer to Figure 1.

Further studies reveal that the spatial distribution rather than the expression level of AQP4 is closely associated with its functions. The loss of perivascular AQP4 is associated with the disappearance of the perivascular astrocyte membrane (Frydenlund et al., 2006), indicating that AQP4 is removed from its functioning sites along with the disassembly of GFAP filaments and glial retraction. Following cerebral ischemia, cortical astrocytes exhibit reduced perivascular AQP4 and unchanged AQP4 protein abundance (Stokum et al., 2015). The loss of AQP4 polarization and the disruption of laminins of the basement membrane disrupt water efflux from the brain while increasing BBB permeability (Steiner et al., 2012). Moreover, in mild focal brain ischemia, these changes occur only in the area of lesion core but not in the penumbra (Steiner et al., 2012). These facts allow us to conclude that AQP4 expression and localization in stroke are correlated with the extent and stage of brain edema formation, which allows different groups of astrocytes to respond to ischemic insults differentially. Figure 2 is a diagrammatic drawing of the astrocytic functional plasticity in ischemic stroke.

## Central Role of Astrocytic Plasticity in Brain Edema Formation

As stated above, astrocytes are not only the major cell type exhibiting cytotoxic edema during ischemia and reperfusion injury, but also a main factor that disrupts BBB integrity. Further analysis reveals that the astrocytic plasticity plays a central role in brain edema formation as detailed below.

### Determining Factor of Ischemic Brain Edema

Astrocytes can extensively modulate the activities of other components of the neurovascular units (Theodosis et al., 2008), and maladaptive alterations of astrocytic plasticity are the major cause of brain edema formation in stroke. Reactive gliosis protects the brain from excessive damage caused by swelling by stabilizing hydromineral balance through absorption of increased extracellular K^+^ (Strohschein et al., 2011) and glutamate (Nagelhus and Ottersen, 2013; Seidel et al., 2016), reducing NH_4_^+^ toxicity and oxidative stress by synthesizing glutamine, antagonizing glutamate toxicity by releasing inhibitory adenosine, taurine and β-alanine (Morán et al., 2001; Parpura et al., 2004), alleviating vasogenic edema by repairing injured BBB, limiting the spreading of inflammation from the infarct core into penumbra, and releasing neuroprotectants (Kawano et al., 2012). In addition, the increased AQP4 level can reduce the osmotic fragility of astrocytes (Gu et al., 2003). However, during prolonged ischemic challenge, astrocytes lose their powers of being an energy provider (e.g., providing metabolic substrates to neurons), a buffer of extracellular chemical and an inhibitor of neuron activity (Marrif and Juurlink, 1999), leading to the malfunction and damage of the entire neurovascular unit. Importantly, inhibiting the activity of AQP4 (Bhattacharya et al., 2013; Yao et al., 2015) and NKCC1 (Su et al., 2002; Yan et al., 2003) significantly reduces ischemic brain edema and the ensuing brain injury while inactivation of GLT-1 worsens damage to the brain (Namura et al., 2002). These facts validate the central role of astrocytes in ischemic brain edema formation.

### Spatiotemporal Correlation

In ischemia, the extent of spatiotemporally differentiated reactive gliosis and the associated RVD is highly correlated with the severity of brain swelling, excitotoxicity, oxidative stress, metabolic disorders, and disruption of the BBB. On the one hand, reactive gliosis, indicated by increasing GFAP and AQP4 expression, is a sign of astrocyte swelling at the early stage of mild stroke and in the penumbra of severe stroke. This gliosis is likely an evolutionary wise selection. It allows the brain, in a situation of a substantial energy deficit in ischemic stroke, to rebalance extracellular K^+^ and glutamate levels through siphoning K^+^ and glutamate uptake, to re-establish neuronal membrane potential and minimize excitotoxic impact at the cost of least energy. However, uncontrolled gliosis can be harmful because it produces high levels of bioactive compounds that are noxious for neuronal cell functions, such as the bioactive free radical nitric oxide (Ghasemi and Fatemi, 2014). In addition, excess gliosis also serves as a trigger of the RVD that could bring additional injury to the neurovascular unit. Thus, despite the protective capacity of gliosis, over-activated astrocytes can worsen the brain edema.

On the other hand, breakdown of this gliosis can lead to degeneration of neural tissues in the lesion core. The reduced volume during the RVD can avoid overload of osmolytes and water in astrocytes, and thus preserve structural integrity of astrocytes. However, the reduced absorption of ion, glutamate and water, decreased volume transmission into the blood in infarct areas as well as the destruction of BBB integrity can seriously disrupt the homeostasis of the whole neurovascular unit, leading to its apoptosis, autophagic cell death and necrosis (Puyal et al., 2013).

### Continuous Involvement

Following the onset of stroke, astrocytes keep changing their structure and function as indicated by the expression of GFAP and AQP4, which determine a conversion of the intracellular edema into extracellular edema in the lesion core while forming a shell of swollen astrocytes at the penumbra. Moreover, overly increased astrocyte volume triggers the RVD wherein the reduced AQP4 could disrupt the adaptive plastic change and dispersion of intracellular volume through the junctional coupling among astrocytes (Nicchia et al., 2005; Mühlfeld and Richter, 2006), thereby leading to the degeneration and even death of astrocytes in the lesion core, promoting vasogenic edema. During the recovery phase, expanded astrocyte processes and newly differentiated glial cells invade the infarct areas to rebuild the neural tissue by cleaning the necrotic tissue, filling in the space and releasing many growth factors (Wasielewski et al., 2012). Thus, astrocytes are continuously involved in the pathogenesis of ischemic brain edema.

## Conclusion

Together with the view presented previously (Verkhratsky et al., 2015), we propose that astrocytes are not only involved in the formation of cytotoxic edema and vasogenic edema but also serve as the central player of the disturbed neurovascular unit in the ischemic brain during edema formation. In this process, astrocytic plasticity exhibits strong spatiotemporal features and continuous involvement in brain swelling, in which, GFAP and AQP4 are the keys to determining the structural and functional plasticity. Further clarification of astrocytic plasticity in ischemic brain edema formation will help to define a therapeutic window of differentially modulating astrocytic plasticity and achieve better prognosis of ischemic brain injury.

## Author Contributions

All authors listed, have made substantial, direct and intellectual contribution to the work, and approved it for publication.

## Conflict of Interest Statement

The authors declare that the research was conducted in the absence of any commercial or financial relationships that could be construed as a potential conflict of interest.
